# Tissue dissection with saline injection: application in thyroid surgery

**DOI:** 10.1093/jscr/rjab500

**Published:** 2021-12-11

**Authors:** Stefanos K Stefanou, Spyros Koulas, Christos K Stefanou, Kostas Tepelenis, Apostolos K Paxinos, Thomas Tsiantis, Vasiliki Galani, Konstantinos Vlachos

**Affiliations:** Department of Surgery, General Hospital “G. Hatzikosta,” Ioannina, Greece; Department of Surgery, General Hospital “G. Hatzikosta,” Ioannina, Greece; Department of Surgery, General Hospital of Filiates, Thesprotia, Greece; Department of Surgery, University Hospital of Ioannina, Ioannina, Greece; Department of Urology, General Hospital of Preveza, Preveza, Greece; Department of Gynecology, University Hospital of Ioannina, Ioannina, Greece; Department of Anatomy-Histology-Embryology, Medical School, University of Ioannina, Ioannina, Greece; Department of Surgery, University Hospital of Ioannina, Ioannina, Greece

**Keywords:** thyroidectomy, hypocalcaemia, parathyroid, tissue dissection, complications

## Abstract

Postoperative hypoparathyroidism is a thyroidectomy complication. The effect of this complication cannot be accurately quantified. The incidence of hypoparathyroidism after total thyroidectomy has high variability in the literature, between 7 and 37%. Data from 78 patients who underwent total thyroidectomy with Tissue Dissection with Solution Injection (TDSI group) from December 2018 to August 2019 were retrospectively reviewed. These patients were compared with 78 patients to whom the technique was not applied (non-TDSI group), and they were treated from January 2018 to September 2018. All thyroidectomies were performed by the same surgeon. The mean duration of a thyroidectomy was 1 hour. The reduction of the incidence of postoperative hypoparathyroidism in the group of patients was applied in respect of the technique of tissue dissection with saline injection. TDSI technique paves the way for further application to other tissues and surgeries.

## INTRODUCTION

A thyroidectomy complication can be postoperative hypoparathyroidism. The effect of this complication cannot be accurately quantified. The measurement of serum parathormone (PTH) immediately after surgery is a sensitive and specific method for assessing the function of parathyroid glands while it can identify patients at risk of hypocalcaemia [1, 2, 3, 4]. The incidence of hypoparathyroidism after total thyroidectomy has high variability in the literature [1]. Part of this variability is related to the variety of methods used to define this complication [5]. Since surgeons are aware that this is a potential risk from thyroidectomy, many patients are treated with either calcium or calcitriol to avoid symptoms preoperatively. While this supplement can help minimize symptoms for patients, this fact makes difficult to determine who has truly transient hypothyroidism and who is not based solely on calcium levels, symptoms or the need for further supplementation.

## MATERIAL AND METHODS

Data from 78 patients who underwent total thyroidectomy with Tissue Dissection with Solution Injection (TDSI group) from December 2018 to August 2019 were retrospectively reviewed. These patients were compared with 78 patients to whom the technique was not applied (non-TDSI group), and they were treated from January 2018 to September 2018. All thyroidectomies were performed by the same surgeon. Mean duration of a thyroidectomy was 1 hour.

### Statistical analysis

We used the Statistical Package for the Social Sciences (SPSS) version 19.0 (SPSS Inc., 2010 Chicago, IL, USA) for our statistical analysis. The level of significance was defined as a probability value *P*<0.05 (*P*, 0.05).

### The surgical technique

**Figure 1:**
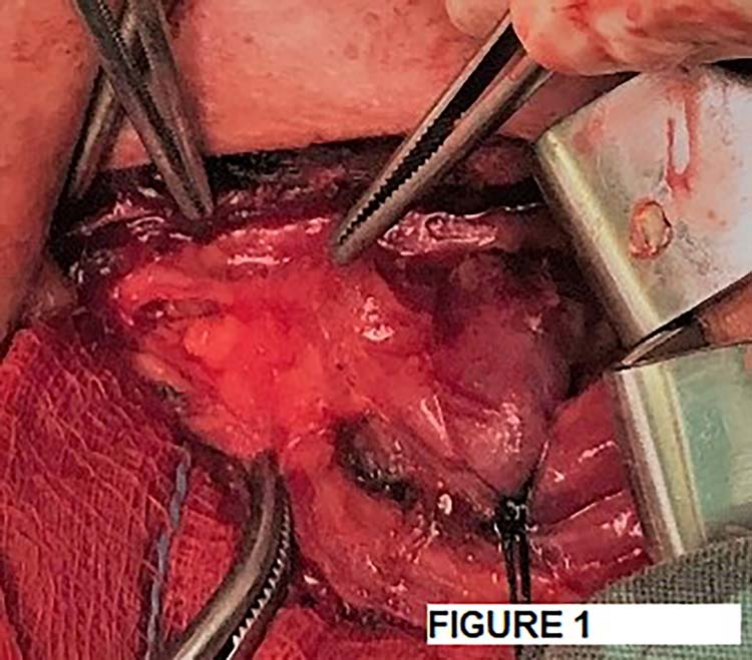
Recognition of the parathyroid gland.

**Figure 2:**
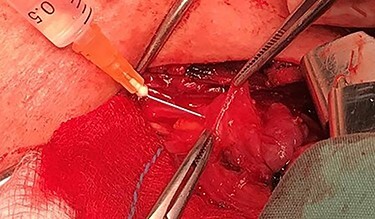
Solution injection with a syringe of 2.5 ml and a needle of 26 gauges, to the loose connective tissue between the thyroid gland capsule and the parathyroid gland.

**Figure 3:**
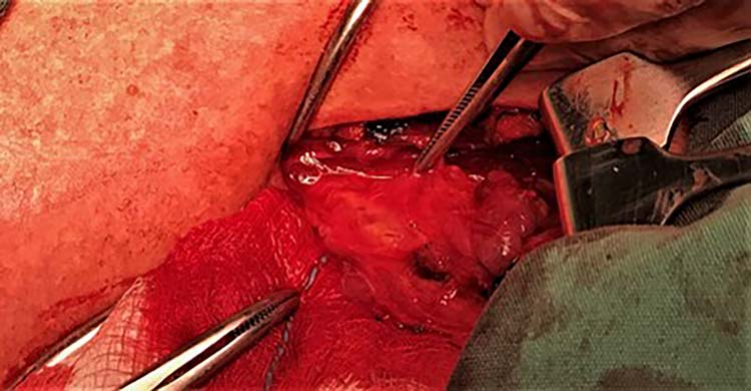
The surgical field after the injection of the solution.

After a careful preparation of the anatomical structures of the neck and exposure of the thyroid gland, we proceeded to recognition of the parathyroid gland (Fig. 1). Then, glycerol solution was injected with a syringe of 2.5 ml and a needle of 26 gauges, to the loose connective tissue between the thyroid gland capsule and the parathyroid gland (Fig. 2). The amount that was administered was such as to produce the desired effect of tissue separation. By injecting the glycerol solution, the tissues swell, the size of the surgical field increases and the anatomical preparation of the parathyroid gland becomes simpler and with minimal parathyroid gland handling. A field like a glass is created, where the vasculature and the limits of the parathyroid gland are distinguished (Fig. 3). After that, a carefully dissection with a Mayo dissection scissor leaves the parathyroid gland intact. This technique is called TDSI.

## RESULTS

As shown in Table 1, there were no significant differences between the two groups (<0.001), which suggested that the two groups are comparable.

**Table 1:** Patients demographics

	TDSI group (*n* = 78)	Non-TDSI group (*n* = 78)	*P* value
Age, mean (SD)	51.79 (11.71)	52.8 (12.92)	<0.001
Sex m:w	29:49	23:55	<0.001
Small cell carcinoma, *n* (%)	5 (6.40)	3 (3.84)	
Goiter disease, *n* (%)	49 (62.80)	58 (74.35)	
Hashimoto, *n* (%)	8 (10.20)	12 (15.38)	
Papillary, *n* (%)	11 (14.10)	2 (2.56)	
Graves, *n* (%)	5 (6.4)	3 (3.84)	

According to Table 2, the TDSI group of patient was less likely to develop transient hypothyroidism postsurgical (*n* = 7, 8.9%) than the group of patient without the application of TDSI technique (*n* = 25, 32.05%, *P* < 0.001). Permanent hypoparathyroidism developed in two patients in the group who did not use the TDSI approach, but no cases of permanent hypoparathyroidism appeared in the TDSI group of patients (*n* = 0, 0%, *n*<0.001). Worth mentioning is the fact that the TDSI patient group had more cases of patients with Graves’ disease, only one patient experienced postsurgical hypocalcaemia with PTH levels after a month >10 pg/ml, unlike the other group of patients with three cases of Graves’ disease, in which all three experienced postsurgical hypocalcaemia and transient hypoparathyroidism.

**Table 2:** Group results

	TDSI group	Non-TDSI group	*P* value
Transient hypoparathyroidism, *n* (%)	7 (8.90)	25 (32.05)	<0.001
Permanent hypoparathyroidism, *n* (%)	0 (0)	2 (2.56)	<0.001
Parathyroid recognized, mean (SD)	3.06 (0.73)	3.22 (0.51)	0.05

## DISCUSSION

The whole idea, of this technique, was inspired by the endoscopic mucosal resection in cancer and polyp cases of gastrointestinal tract. The idea of expanding the site of excision and the protection of adjacent tissues is something that can be applied to other organs.

First of all, we have chosen glycerol as the injected solution because glycerol is a hypertonic solution consisting of 10% glycerin and 5% fructose in an NS solution. By injecting the glycerol solution, we achieve the following: firstly, the space between the parathyroid gland and the thyroid gland is expanding and can be easily detached. Second, a gel-like capsule shapes around the parathyroid gland, protecting it from manipulations that could harm the gland's structure and vascularization, as well as thermal trauma. According to Sumiyoshi *et al*. [6], the glycerol group maintained a significant longer-lasting submucosal elevation than the NS group. There were no problems involving histopathologic tissue damage to the specimens due to the injection in either group. The use of glycerol was shown to safely increase en bloc resections in this study. Glycerol is relatively inexpensive and readily available and is considered superior to NS.

We also observed that it is possible to exploit the loose connective tissue between the thyroid gland and the parathyroid gland. Parathyroid glands are small glands and the difficulty in identifying them is related to the surrounding fat and lymph nodes. This requires careful surgical preparation of the glands. Knowledge of the anatomy, suspicion, recognition and anatomic surgical preparation are the 4-fold of the reduction of postoperative hypothyroidism. Manipulations in the area of the parathyroid gland can transiently affect blood flow to the gland or reduce vein outflow. The end-branch blood flow to the parathyroid gland is mainly oblique in the medial direction. Therefore, mobilization and maintenance of the vessels that are laterally to the gland are essential to prevent recurrence. The localization and intact preparation of the parathyroid gland are not sufficient to prevent hypocalcaemia. The aim of the presented technique is to increase the size of the surgical field and to exploit the loose connective tissue between the capsule of thyroid gland and the parathyroid gland.

In cases of thyroid cancer, it is safe to use TDSI technique because injection is performed at the loose connective tissue and not at the thyroid capsule. However, further investigation is needed.

## CONCLUSION

TDSI technique paves the way for further application to other tissues. At thyroid surgery, the incidence of postsurgical hypocalcaemia and hypoparathyroidism is reduced. Knowledge of anatomy, suspicion, recognition and anatomic surgical preparation are the 4-fold of the reduction in the rate of postoperative hypothyroidism. However, further study is required to fully understand.

## References

[1] McCullough M, Weber C, Leong C, Sharma J. Safety, efficacy, and cost savings of single parathyroid hormone measurement for risk stratification after total thyroidectomy. Am Surg 2013;79:768–74. 23896242

[2] Chow TL, Choi CY, Chiu AN. Postoperative PTH monitoring of hypocalcemia expedites discharge after thyroidectomy. Am J Otolaryngol 2014;35:736–40. 10.1016/j.amjoto.2014.07.006 25091178

[3] Fahad Al-Dhahri S, Al-Ghonaim YA, Sulieman TA. Accuracy of postthyroidectomy parathyroid hormone and corrected calcium levels as early predictors of clinical hypocalcemia. J Otolaryngol Head Neck Surg 2010;39:342–8. 20642997

[4] Rivere AE, Brooks AJ, Hayek GA, Wang H, Corsetti RL, et al. Parathyroid hormone levels predict posttotal thyroidectomy hypoparathyroidism. Am Surg 2014;80:817–20. 25105405

[5] Edafe O, Antakia R, Laskar N, Uttley L, Balasubramanian SP. Systematic review and meta-analysis of predictors of post-thyroidectomy hypocalcaemia. Br J Surg 2014;101:307–20. 24402815 10.1002/bjs.9384

[6] Sumiyoshi T, Fujii T, Sumiyoshi Y, et al. Injected substances to the submucosa in endoscopic mucosal resection: glycerin solution versus normal saline solution [abstract]. Gastrointest Endosc 2002;55:AB110.

